# What is the role of lifestyle behaviour change associated with non-communicable disease risk in managing musculoskeletal health conditions with special reference to chronic pain?

**DOI:** 10.1186/s12891-015-0545-y

**Published:** 2015-04-13

**Authors:** Elizabeth Dean, Anne Söderlund

**Affiliations:** Department of Physical Therapy, Faculty of Medicine, University of British Columbia, Vancouver, V6T 1Z3 Canada; School of Health, Care and Social Welfare, Mälardalen University, Box 883, Västerås, SE- 721 23 Sweden

**Keywords:** Exercise, Physical therapy outcomes, Lifestyle behaviour change, Knowledge translation

## Abstract

**Background:**

Other than activity and exercise, lifestyle practices such as not smoking and healthy nutrition, well established for preventing and managing lifestyle-related non-communicable diseases (i.e., heart disease, cancer, hypertension, stroke, obstructive lung disease, diabetes, and obesity), are less emphasized in the physical therapy guidelines for addressing chronic pain, e.g., back pain. This state-of-the-art review examines the relationships between lifestyle behaviours and musculoskeletal health, with special reference to chronic pain, and their clinical and research implications.

**Discussion:**

A state-of-the-art review was conducted to synthesize evidence related to lifestyle factors (not smoking, healthy diet, healthy weight, optimal sleep and manageable stress, as well as physical activity) and musculoskeletal health, with special reference to chronic pain. The findings support that health behaviour change competencies (examination/assessment and intervention/treatment) may warrant being included in first-line management of chronic pain, either independently or in conjunction with conventional physical therapy interventions. To address knowledge gaps in the literature however three lines of clinical trial research are indicated: 1) to establish the degree to which traditional physical therapy interventions prescribed for chronic pain augment the benefits of lifestyle behaviour change; 2) to establish the degree to which adopting healthier lifestyle practices, avoids or reduces the need for conventional physical therapy; and 3) to establish whether patients/clients with healthier lifestyles and who have chronic pain, respond more favourably to conventional physical therapy interventions than those who have less healthy lifestyles.

**Summary:**

Lifestyle behaviour change is well accepted in addressing lifestyle-related non-communicable diseases. Compelling evidence exists however supporting the need for elucidation of the role of negative lifestyle behaviours on the incidence of chronic pain, and the role of positive lifestyle behaviours on its incidence and effective management. Addressing lifestyle behaviour change in patients/clients with chronic pain, e.g., back pain, as a first-line intervention might not only constitute a novel approach, but also reduce the socioeconomic burden related to chronic pain as well as non-communicable diseases.

## Background

Chronic pain, e.g., back pain, is one of the most common conditions seen by physical therapists and physicians. Clinical practice guidelines for patients/clients with chronic back pain include conventional physical therapy combined with back care and ergonomic education [1]. Chronic back pain is known to be associated with inactivity as both a cause and an effect. In addition, musculoskeletal problems including the presence of chronic pain and exercise incapacity is associated with several other lifestyle behaviour practices that are associated with lifestyle-related non-communicable diseases [2]. These include smoking, unhealthy diet, unhealthy weight, poor sleep, and unmanageable stress. Despite mounting evidence to support lifestyle behaviour change as an intervention to prevent and manage chronic pain, multidisciplinary team approaches appear more likely to focus on the adequacy of patents’ movement behaviours to achieve physical and functional recovery [3].

To clarify the scope of literature related to lifestyle behaviours and chronic pain, a narrative state-of-the-art review was conducted. To our knowledge no review of the literature exists that examines these relationships specifically. Our first aim was to synthesize evidence related to the relationships among common lifestyle behaviours including smoking, diet, body weight, sleep quality, and stress as well as physical activity, and musculoskeletal signs and symptoms particularly pain and its antecedents, e.g., inflammation. The evidence we report is based on literature representing a breadth of scholarly paradigms including clinical trials, cross sectional population based studies, expert narrative reviews, randomized controlled clinical trials, and systematic reviews. Databases and search tools included the Cochrane Database of Systematic Reviews to ensure that no reviews had been conducted on the topic. Studies were retrieved from the following databases: MEDLINE (OvidSP), EMBASE (OvidSP), and CINAHL (EBSCO). Appropriate subject headings and relevant keywords were used to thoroughly describe the target population of people’s musculoskeletal health/signs and symptoms with special reference to pain and smoking/not smoking; unhealthy nutrition/healthy nutrition; sedentary behaviour/physical activity and exercise; sleep deprivation/healthy sleep; and anxiety and stress/good mental health. As a preliminary step, we chose to report and synthesize the literature broadly given the breadth of research paradigms and limited controlled trials amenable to systematic review. We focused the search on articles that related to chronic pain and its correlates, including back pain where literature existed. Our second aim was to examine the level of support for integrating lifestyle behaviour change into the clinical management of chronic pain and its correlates, and to propose lines of research investigation to address gaps in the literature.

## Discussion

### Health behaviours and relationship to pain

#### Smoking

Smoking is not only harmful to general health and exercise capacity, but also to the musculoskeletal system [4-6]. A positive relationship exists between years smoked and bone thinning and fractures, which is also observed in second-hand smokers. Smoking delays healing and increases complications from fractures and trauma. Bone loss is exacerbated with high caffeine and alcohol consumption which often accompany smoking [7].

Smoking has been associated with local inflammation affecting the musculoskeletal system. In a Finnish study of almost 6,000 participants, smoking was identified as an independent risk factor for epicondylitis [8]. Further studies are needed to establish whether this relationship extends to smokers with other types of acutely painful conditions as well as pain that progresses to chronicity.

The relationship between smoking and pain has been well established. Smoking aggravates the progression of back pain and arthritis [4]. Smokers have been reported to have a lower pain threshold and experience more pain that non-smokers and former smokers [9,10]. Both smoking early in life and over many years increase the risk of experiencing frequent pain over one’s life [4].

Given the deleterious effects of smoking on musculoskeletal health, quitting smoking improves musculoskeletal health and functional capacity in several ways. In addition to the well-documented improvement in cardiovascular and pulmonary function, bone density is better preserved over time, and vascular perfusion of muscle and bone is maximized for nourishment, healing and repair, and augmenting systemic and local immune and anti-inflammatory responses [11]. As well as improving exercise endurance, quitting smoking lowers rate of bone loss and fractures, but may require several years to reduce to rates observed in non-smokers [12]. In addition, outcomes after orthopaedic surgery are improved in smokers who have quit, e.g., faster wound healing, and reduced complications and hospital stays [13], hence faster recovery of functional capacity.

#### Unhealthy diet and weight

Exercise capacity is compromised by diet in terms of availability of micro and macro nutrients, and by body mass, in terms of biomechanics and energy cost. Optimal nutrition and physical activity during the bone-building years of childhood are singularly important in determining bone health over the course of one’s life. Bone demineralization and conditions of osteopenia and osteoporosis are highly associated with nutrition and activity in adults. Low bone mineral density is also a risk factor for cardiovascular disease which in turn has implications for the targeted prescription of exercise in the management of chronic back pain in such a cohort [14].

A key index of bone health with respect to mass and density is calcium balance [15]. Western lifestyle practices contribute to calcium negative balance and bone demineralization. Guidelines for maintaining calcium balance frequently focus on calcium loading, e.g., calcium supplementation rather than reducing calcium loss. Although a requisite amount of calcium is required for healthy physiological functioning, the depletion of calcium through sedentary living, caffeine and alcohol consumption, smoking and possibly animal protein consumption, is less well appreciated and addressed clinically, despite these relationships being well documented [16,17].

Bone loading secondary to excess body mass does not protect a person against bone demineralization and fractures, in fact may contribute to bone fragility. High body mass impairs the constituents of bone that are most important for bone strength and fracture protection [18]. Excessively-high body mass limits a person’s functional capacity, and compensatory maneuvers used to augment mobility by people who are overweight or obese can contribute to musculoskeletal strain.

Excessive body mass has been unequivocally associated with chronic pain as well as ischaemic heart disease, type 2 diabetes mellitus, and high blood pressure. Lifestyle behaviour change has been advocated for people who are obese to not only improve health outcomes but also improve mobility and quality of life [19,20].

Chronic lifestyle-related conditions including obesity are associated with chronic systemic low-grade inflammation (CSLGI) [21,22]. The degree to which CSLGI can lower a person’s threshold for local inflammation is an interesting and relevant clinical question. The work of Shiri et al. [8] supports this hypothesis. They reported that obesity is an independent risk factor for local inflammation such as epicondylitis.

Obesity has been associated with a range of musculoskeletal conditions that impact functional capacity. In addition to back pain [23,24], people who are obese are more prone to hyperuricemia and gout [25], and osteoarthritis [26]. In addition, body mass and pain threshold have been reported to be inversely related [27,28].

#### Inactivity

Sedentary lifestyles are well known to be associated with non-communicable diseases including heart disease, high blood pressure and stroke, type 2 diabetes mellitus, and some cancers which explains why lifestyle-related conditions tend to cluster [29]. With respect to its impact on musculoskeletal health, sedentary living is independently associated with back problems [30]. In addition, inactivity has been associated with joint degeneration due to reduced synovial fluid production to protect joint surfaces [31].

Even when back pain is acute, tolerable physical activity is now advised as standard practice, as opposed to former recommendations of bed rest [32]. People with chronic conditions, including back pain, walk less and are generally less physically active than those without pain. This phenomenon could be confounded by fear of pain and pain avoidance behaviour [33]. McDonough and colleagues [34] recently reported that people with low back pain can not only increase their walking capacity with reduced disability and pain with a pedometer-driven walking program, but also with no ill effects.

#### Poor sleep

People in western cultures have been reported to be largely sleep deprived which impacts both their physical and cognitive functioning [35,36]. Since the turn of the 20th century, the average night’s sleep has been reduced by about two hours. The optimal number of hours of quality sleep (characterized by periods of REM sleep) ranges from 8 to 10 however people are more likely to report sleeping for less than seven hours nightly. Sleep deprivation studied largely in shift workers is well known to be associated with increase illness and injury rates, and is independently linked to high blood pressure and heart disease [37].

Sleep deprivation can exist independently of the patient’s presenting complaint to the physical therapist, e.g., back pain, or can result from it. Recent reports show that some 50 per cent of people with low back pain report insomnia [38,39]. Insomnia is associated with fatigue, cognitive disturbance, mood disturbance, anxiety and depression [40]; thus, symptoms that can confound the presentation of chronic pain.

People with pain not only sleep less well, but when their sleep is improved, they report less pain [37]. Further, insomnia has been shown to lower a person’s pain threshold and tolerance [41,42], and this is true for people without a health condition [43]. The best predictors of insomnia severity even when pain intensity is controlled [44] are affective pain rating and health anxiety [44].

Tissue injury related to the stress of sleep deprivation has been proposed to be mediated by impaired immune response and potentially free radical production [45]. Findings from animal studies support that regular REM sleep is essential to minimize free radical production and consequent tissue irritation and damage [46]. Healing may be impaired due to CSLGI, an index of an overwhelmed immune system and impaired immune response, and is independently associated with chronic sleep deprivation [45].

#### Stress

Mental health directly impacts musculoskeletal health and functional capacity. The relationship between musculoskeletal health and mental health is bidirectional. People with mental health issues are more susceptible to physical health issues including musculoskeletal problems [47], and people with musculoskeletal problems often experience a range of mental health issues including anxiety, unmanageable stress, and depressive symptoms [48]. Such mental health issues can reduce pain threshold and further increase its impact on physical capacity and disability [49].

People with mental ill health have poorer lifestyle practices than people without such challenges, e.g., smoke more, eat less well, are more overweight/obese, are less active, sleep less well, and are more troubled by stress and anxiety [50,51].

Conversely, people with chronic musculoskeletal complaints particularly pain, are well known to experience mental health issues including anxiety, stress and depressive symptoms [52,53]. And further, depressive symptoms negatively impact the course of low back pain at least in the acute stage [54]. Not inconceivably, this is likely true for the chronic stage of pain. Thus, monitoring potential depressive symptoms for at least six weeks after shoulder pain onset has been recommended [54].

Implementing strategies to improve a patient’s mental health, be it compromised independently or secondarily to the presenting musculoskeletal problem, may improve his or her functional self-efficacy, resilience, perceived control, and resources to manage his or her presenting complaint in the short and long terms, thereby augmenting therapeutic outcome and recovery of the specific complaints [55].

Physical activity and exercise programmes have been recommended for people with mental health issues. People who are active and exercise more regularly report better mental health, i.e., less anxiety and depressive symptoms, than those who are not active nor exercise [56]. Increasing general physical activity levels of people with chronic pain such as those with back pain therefore may help offset the effects of the pain on mental health, reduce the pain, independently of any improved mental health secondary to an improvement of the pain in response to conventional management.

### Clinical implications

Lifestyle behaviour change warrants being considered in musculoskeletal care including chronic pain management in that common lifestyle behaviours, often associated with conditions such as ischaemic heart disease, impact musculoskeletal health and functional capacity. Although we have identified research themes that are indicated to elucidate the implications of the state-of-the-art literature we report, for clinical practice there is sufficient evidence to recommend at least assessing a patient’s lifestyle behaviours and to consider their impact on musculoskeletal signs and symptoms. Although further research will elucidate the role of lifestyle behaviour change in the management of musculoskeletal signs and symptoms such as chronic pain, healthy living practices are additionally beneficial in reducing chronic disease risk as well as improving quality of life in a dose dependent manner [57,58].

### Research implications

The literature related to lifestyle influences on musculoskeletal health including pain and its correlate inflammation has been largely based on cross sectional studies and correlational analysis. Long-term prospective studies however are challenging given ethical considerations regarding depriving patients of interventions known to benefit them, specifically, lifestyle behaviour change. Intervention studies however can be designed to isolate which lifestyle behaviour changes are most effective in preventing and mitigating pain, thereby augmenting exercise capacity, in which patients at what time and how these benefits may augment the benefits of physical therapy. Four primary themes are shown in the Appendix. Such studies would help to inform comprehensive patient examination and assessment with respect to lifestyle behaviours for physical therapists, as well as behaviour change strategies they can expediently integrate into practice. Long-term cross sectional studies will help establish differences in the rate of recurrence of symptoms and need for medication and surgery over time, in patients who are treated with physical therapy alone, lifestyle behaviour change alone, or some combination. Post hoc data analysis of clinical outcomes of physical therapy interventions stratified based on health status and lifestyle variables of the patients is another means of examining the role of lifestyle behaviours and practices on chronic pain and treatment outcomes including physical therapy.

Finally, elucidation of lifestyle factors such as smoking, nutrition and body mass, activity level, sleep status and stress is needed to not only prevent or reduce the need for medication for musculoskeletal health conditions, but also to understand interaction effects of healthy living and patients’ drug responses. For example, Wilson et al. [59] recently argued for the inclusion of lifestyle behaviour change in antihypertensive drug trials, or at least control of lifestyle factors to tease out these relationships. On the basis of such evidence, core competencies in health behaviour change have been proposed for all established health professions [60]. Elucidation of the relationships between lifestyle behaviours and musculoskeletal signs and symptoms including pain will better inform the role of medications and need for surgery.

## Summary

Lifestyle practices including smoking, poor nutrition (consuming a pro-inflammatory standard western diet), unhealthy body weight, inactivity, poor sleep, and unmanageable stress, can independently or in combination confound musculoskeletal signs and symptoms including chronic pain such as back pain, inflammation and further functional incapacity. Sufficient evidence exists to support physical therapists considering incorporating lifestyle behaviour assessment in patients with chronic musculoskeletal signs and symptoms including chronic pain, and consider lifestyle factors as potential confounders to the patient’s presentation. The physical therapist needs to make an independent case-by-case decision regarding whether lifestyle behaviour change is warranted. Compared with the effects of conventional physical therapy interventions, the benefits of lifestyle behaviour change on musculoskeletal outcomes may take weeks or months to demonstrate. Nonetheless, physical therapists are committed to exploiting best evidence-based practice, particularly non-pharmacological interventions, and the health of the individual overall.

Well-controlled prospective and retrospective studies are needed to establish the relative contributions of adverse lifestyle behaviours to musculoskeletal impairments including chronic pain for a given individual, and establishing the best behaviour change strategies for that individual to prevent or remediate chronic pain, prevent pain recurrence, and maximize functional capacity.

## Appendix

### Evidence-based themes proposed for clinically-relevant research to examine relationships among lifestyle behaviours and musculoskeletal health

#### Theme 1

The degree to which western lifestyle practices compromise musculoskeletal health and contribute to the pain experience and related exercise incapacity:

- smoking vs. not smoking
- standard western diet vs. Mediterranean-type diet
- unhealthy weight vs. healthy weight
- sedentary lifestyle vs. physically active lifestyle
- suboptimal sleep vs. healthy restorative sleep
- and chronic unmanageable anxiety/stress vs. manageable anxiety/stress

#### Theme 2

Compared with conventional physical therapy for chronic pain, the degree to which maximizing healthy living practices:

- prevents chronic pain; and minimizes its risk and impact, and the need for drugs/surgery
- differentially influences these outcomes
- maximizes long-term outcomes including lifelong health and wellbeing associated with minimal or the absence of chronic pain

#### Theme 3

With respect to the physical therapy management of chronic pain, e.g., back pain, to elucidate the outcomes of:

- conventional physical therapy alone
- lifestyle behaviour change alone
- some combination of the two approaches including the degree to which lifestyle behaviour changes augment the outcome of conventional physical therapy including pain reduction and increasing functional capacity, i.e., which health behaviours for which patients, at what time, and under what circumstances?

#### Theme 4

Evidence-informed revision of clinical practice guidelines for the physical therapy management of chronic pain, e.g., back pain, based on the literature on lifestyle risk factors commonly associated with non-communicable diseases, and their relationships to musculoskeletal health.
